# LPS-Induced proNGF Synthesis and Release in the N9 and BV2 Microglial Cells: A New Pathway Underling Microglial Toxicity in Neuroinflammation

**DOI:** 10.1371/journal.pone.0073768

**Published:** 2013-09-09

**Authors:** Li Duan, Bei-Yu Chen, Xiao-Long Sun, Zhuo-Jing Luo, Zhi-Ren Rao, Jing-Jie Wang, Liang-Wei Chen

**Affiliations:** 1 Institute of Neurosciences, The Fourth Military Medical University, Xi’an, China; 2 Department of Orthopedics, Xijing Hospital, The Fourth Military Medical University, Xi’an, China; 3 Department of Neurology, Xijing Hospital, The Fourth Military Medical University, Xi’an, China; 4 Department of Gastroenterology, Tangdu Hospital, The Fourth Military Medical University, Xi’an, China; Inserm U837, France

## Abstract

**Purpose:**

While aberrant activation of microglial cells was evidently involved in neuroinflammation and neurotoxicity in the neurodegenerative diseases such as Alzheimer’s and Parkinson’s disease, objective of study was to address if activated microglias deliver their effect by releasing pro-neurotrophins.

**Materials and methods:**

By *in vitro* culture of N9 and BV2 cell lines and lipopolysaccharide (LPS) stimulation model, generation and release of proNGF, proBDNF and MMP-9 was studied in the activated microglial cells by immunocytochemistry, western blotting and bioassay methods.

**Results:**

Activation of microglial cells was observed with obvious increasing iba1-immunoreactivity following LPS stimulation in cell culture. Synthesis and up-regulation of proNGF protein significantly occurred in N9 and BV2 cells 12h-48h after LPS exposure, whereas no significant changes of proBDNF and MMP9 were observed in these microglial cell lines with LPS insult. More interestingly, extracellular release or secretion of proNGF molecule was also detected in culture medium of N9 cells after LPS stimulation. Finally, bioassay using MTT, Hoechst/PI and TUNEL staining in SH-SY5Y cells further confirmed that proNGF treatment could result in apoptotic cell death but it did not significantly influence cell viability of SH-SY5Y cells.

**Conclusions:**

This *in vitro* study revealed LPS-stimulated proNGF synthesis and release in activated N9/BV2 microglial cell lines, also suggesting that proNGF may appeal a new pathway or possible mechanism underlying microglial toxicity in the neuroinflammation and a potential target for therapeutic manipulation of the neurodegenerative diseases.

## Introduction

Accumulating evidence has shown that the reactive glial cells or aberrant activation of glial cells are crucially involved in neuroinflammation and neuronal injury in several neurodegenerative disorders such as Alzheimer’s disease (AD), Parkinson’s disease (PD) and amyotrophic lateral sclerosis (ALS) [1–3], but it still remains to address how those activated glial cells deliver specific neurotoxic effects. Although roles of many pro-inflammatory cytokines such as the interleukins and tumor necrosis factor alpha were demonstrated in pathological events [2,3], abnormal switch or imbalance of neurotrophin function might also implicated in the glial cell-mediated neurotoxicity, particularly in injury or disease conditions [4–6]. With expectation of neurotrophic therapy for the neurodegenerative diseases, it is known that various neurotrophins such as nerve growth factor (NGF) and brain-derived neurotrophic factor (BDNF) plays important roles in maintaining neuronal cell survival, differentiation and neurite growth of the central nervous system (CNS) [7,8]. Unexpectedly, however, it is also identified that proforms of several neurotrophins could induce neuronal cell death or loss by preferential binding to p75NTR-sortilin receptor and triggering apoptosis-related signaling in aging state and diseases [4–6]. Studies have suggested deficiency in mature neurotrophins, abnormality in neurotrophic support or imbalance in proform of neurotrophins and mature neurotrophins might possibly constitute one major cause in pathogenesis and disease progression of aforementioned neurodegenerative diseases in human beings [9–14].

In the CNS, neurotrophins such as NGF and BDNF are initially synthesized as pro-neurotrophins, that are then cleaved to release mature C-terminal forms. The proforms of neurotrophins such as proNGF and proBDNF, preferentially bind to p75NTR-sortilin receptor whereas mature neurotrophins are preferred ligands for Trk receptors. While signals emanating from Trks support neuronal survival, cell growth and synaptic strengthening, the proNGF-p75NTR-sortilin signaling can induce apoptosis, attenuate growth and weaken synaptic signaling [15–22]. Accumulating evidences have indicated that p75NTR-sortilin signaling triggered by abnormality or imbalance of proNGF/NGF might be involved in the glial-neuronal interaction, degenerative loss of motor neurons or cholinergic neurons, disease onset or progression in AD, PD and ALS [9–14]. However, it still remains a critical question if proforms of neurotrophins can be synthesized and directly secreted from the activated glial cells. By applying N9 and BV2 cell culture and lipopolysaccharide (LPS) exposure model in this study, therefore, we examined dynamic patterns of activated microglial cells and revealed LPS-induced proNGF synthesis and release from these activated microglial cells.

## Methods

### Cell culture of N9 and BV2 microglial cells

The murine N9 and BV2 microglial cell lines were used in this study. The N9 cell line (kindly provided by Dr. H. Yang, Institute of Neuroscience, Fourth Military Medical University, China) was prepared by Righi et al [23] through immortalization of E13 mouse embryonic brain cultures with the 3RV retrovirus carrying an activated v-myc oncogene, while BV-2 immortalized murine microglial cell line (kindly provided by Dr. M. Shi, Department of Neurology, Xijing Hospital, Fourth Military Medical University, China) was generated by Blasi et al [24] through infecting primary microglial cell cultures with a v-raf/v-myc oncogene carrying retrovirus. For cell culture, briefly, N9 cells and BV2 cells were respectively seeded in 75cm^2^ flasks in density 0.5-1×10^6^/ml and cultured in 15ml high glucose DMEM medium(Hyclone, USA) supplemented with 10% fetal calf serum (Hyclone, USA) and 100 U/ml penicillin/streptomycin (Hyclone, USA). After culture in a humidified 5% CO2/95% air incubator at 37°C for about 5-7 days, the cells were first allowed to grow in 70-80% confluence and processed for LPS exposure experiments. For the purpose to eliminate any interference effect of serum proteins or factors, cultured cells were replaced with serum-free medium after three rinses of D–PBS. Cell cultures were then subjected to exposure of LPS at 100ng/ml concentration for distinct time-points, i.e. 0h, 12h, 24h and 48h, which are processed as below for cell samples.

$\begin{array}{cc} & \text{Serum-free 1h}\to \text{PBS 12h Cell Sample}\\ \text{N9/BV2 cells} & \text{Serum-free 1h}\to \text{LPS 12h Cell Sample}\\ \text{(70-80\% confluence)} & \text{ Serum-free 1h}\to \text{LPS 24h Cell Sample}\\ & \text{Serum-free 1h}\to \text{LPS 48h Cell Sample} \end{array}$

After LPS exposure, these different cultures were subjected to immunocytochemical and western blotting detection of proNGF, NGF, proBDNF, BDNF and MMP-9 synthesis in the cells. Besides, to detect direct release of proNGF extracellularly, the 15 ml conditioned medium was also collected from LPS 24h and 48h groups, centrifuged at 800g for 5 min to precipitate non-adherent cells. The supernatant was collected and filtered through a 0.2μm membrane to remove any cell debris. The filtrate was concentrated to 500μl using Amicon Ultra-15 Centrifugal Filter Devices (Millipore). Then the 500 μl filtrate was further concentrated to about 50μl using Amicon Ultra-0.5 Centrifugal Filter Devices (Millipore). About 25μl of the final filtrate was separated by 12% SDS-PAGE and the remaining proteins were detected by western blot protocol as following.

### Immunofluorescence and laser scanning confocal microscopy

Immunofluorescence and laser scanning confocal microscopy were done to reveal localization of proNGF, proBDNF, and MMP-9 in the cultured N9 and BV2 cells. Briefly, the cells were first fixed with 4% paraformaldehyde for 10 min and fallowed with three washes of 0.01M phosphate buffered saline (PBS). Then cells were incubated with 10% donkey serum-containing blocking solution for 30min at room temperature, and followed by incubation of primary antibody solution containing 10% donkey serum, 0.3% triton X-100 in 0.01M PBS at 4°C for 24h, i.e. rabbit anti-proNGF (Sigma, P5498, 1:800 dilution), rabbit anti-NGF (Sigma, N6655, 1:800 dilution), rabbit anti-proBDNF (Sigma, P1374, 1:800 dilution), rabbit anti-BDNF (Santa Cruz, Sc-20981, 1:100 dilution), rabbit anti-MMP-9 (Sigma, SAB4501896, 1:500 dilution), respectively. After three washes with PBS, the cells were then incubated with Alexa Fluor-488 conjugated donkey rabbit IgG (1:500, Molecular Probes) for 4hs at room temperature. With DAPI nuclear counterstaining for 10 min, the coverslips were mounted with Fluorescence-preserving VECTASHIELD Mounting medium (Vector, H-1000) and examined under confocal microscope. For control experiments, primary antibody was substituted with normal rabbit serum in immunostaining. Immunoreactive or positive cells were not found in these control staining samples and specificity of antibodies used was also indicated in the manufacture’s data-sheet.

### Western blotting

Western blotting was performed to quantify protein expression of proNGF, NGF, proBDNF, BDNF and MMP-9 in N9 and BV2 cell culture in a standard protocol. Briefly, fresh cell samples were collected after soft typsin digestion and homogenized at 4°C in 5 volumes of extraction buffer [50mMTris (pH7.4), 150mM NaCl, 1% NP-40, 0.5% sodium deoxycholate, 0.1% SDS, and protease inhibitor cocktail (Complete, Roche Diagnostics)] using a hand-held homogenizer. Cell lysates were incubated at 4°C with constant shaking for 10 min and were centrifuged at 13,000 g for 15 min at 4°C. Protein concentration was determined by BCA assay (Beyotime, China). After measurement of total protein amount, supernatant was mixed with four volumes of protein loading buffer, boiled for 5min at 99°C and stored at -20°C. Proteins were separated in SDS-PAGE gels (10% or 12%) and transferred onto PVDF membranes (millipore). Membranes were blocked with 5% non-fat milk/PBST (1% Tween-20, PBS) for 1 h, and incubated with the following antibodies proNGF, NGF, proBDNF, BDNF, MMP-9 (diluted in PBST) at 4°C overnight. After washing in PBST, membranes were incubated with appropriate HRP-conjugated secondary antibody (1:2000 in PBST) at room temperature for 1 h. For detection, an enhanced chemiluminescence (ECL) detection system (CWBio, China) was used according to the manufacturer’s instructions. As a control for protein loading and transfer, membranes were stripped, incubated with the mouse monoclonal antibody against actin (ZSGB-Bio, China) and revealed as described. Images were digitally acquired and analyzed using ChemiScope System (Clinx). Besides, western blotting was also performed to detect extracellular release of proNGF in culture medium of N9 cells in aforementioned protocol.

In addition, trypan blue staining was further performed in cell culture in order to exclude the release of proteins due to cells cracking. The adherent cells were trypsinised and combined with non-adherent cells to assess cell viability by trypan blue exclusion test. Trypan blue dye (Sigma) was diluted in PBS (0.4% w/v) and added in cell suspension, cell count was made under a microscope. The ratio of total viable cells to the sum of viable and dead cells was used to reflect cell viability changes among different cell groups. For control experiment, the presence of actin was also detected by western blot in both culture medium and cell lysates.

### SH-SY5Y cell culture, MTT, Hoechst/PI and TUNEL staining

The bioassay was finally performed in human neuroblastoma SH-SY5Y cells to show any effect of recombinant human proNGF on cell viability and cell death by MTT, Hoechst/PI and TUNEL staining. The SH-SY5Y cell line was purchased from Rio de Janeiro Cell Bank (BCRJ, Brazil) and property of SH-SY5Y cells was well characterized by Biedler et al [25]. For cell culture, SH-SY5Y cells were grown in DMEM/F-12 supplemented with 10% FBS (v/v), penicillin (100 IU/ml), and streptomycin (100 mg/ml) in humidiﬁed 5% CO2/95% air at 37°C. SH-SY5Y cells were cultured at a density of 1x10^5^ cells/ml on 96-well plates. When cells were at 80% conﬂuence, stock solution of recombinant human proNGF (rhproNGF, SinoBio, C078, Shanghai, China) was added the free serum cultural medium at final concentration of 0,1, 2.5, 5, 10, 20, 40ng/ml and kept for 24 h. Cell samples were respectively processed for MTT assay, Hoechst/PI and TUNEL staining to detect cell viability and cell death or cell apoptosis. Besides, western blot was also performed to detect expression of p75NTR and soritlin, which mediate proNGF-triggering apoptosis signaling and cell death [14,16,20], in control and LPS exposure conditions, and rabbit anti-p75NTR (Sigma, 1:800 dilution) and rabbit anti-sortilin (Sigma, 1:800 dilution) were used for immunoblotting in an ordinary protocol same as above.

MTT assay was performed to quantify viability of SH-SY5Y cells after proNGF treatment, MTT [3-(4,5-dimethyl)-2,5-diphenyl tetrazolium bromide] stock solution (5mg/mL) was added to the incubation medium in the wells at a final concentration of 0.2 mg/mL and were kept for 4h at 37°C in a humidified 5% CO2 atmosphere. Culture medium was then removed and formazan crystals in the cells were solubilized with dimethyl-sulfoxide (DMSO) by plate shaking for 30 min. The absorbance was measured at 570 nm using a microplate reader (Infinite M200 PRO TECAN, UK). The optical density of the formazan generated in the control cells was considered to represent viability.

Hoechst/PI staining was performed to detect any cell damage of SH-SY5Y cells after proNGF treatment. The cell samples of 0ng/ml, 10ng/ml and 40ng/ml groups were incubated for 20 minutes at RT with Hoechst 33342 (10μg/mL; Molecular Probes) and propidium iodide (PI, 1:1000). PI could become permeable due to cell damage and only stain nuclei of SH-SY5Y cells with damage Images were acquired using a fluorescence microscope (Olympus) and number of dying cells (PI-positive) was counted and expressed as a percentage of total cells stained with Hoechst 33342.

The terminal deoxynucleotidyl transferase dUTP nick end labeling (TUNEL) staining was further carried out to evaluate apoptotic cell death of SH-SY5Y cells with proNGF treatment by TUNEL assay (Roche, Mannheim, Germany). Cells were fixed by paraformaldehyde for 20min and followed by rinse with 0.01M PBS, permeabilized in 0.25% Triton X-100 for 30min at room temperature, and incubated with terminal deoxynucleotidyl transferase buffer for 1h at 37°C in a humidified chamber. Afterwards, cells were incubated in terminal buffer (300mM sodium chloride and 30mM sodium citrate) for 10 min and in 0.01M PBS for 5min. Incubation with fluorescein Avidin D (1:100; Vector, USA) was performed for 1h, followed by nuclei counterstaining with DAPI (Sigma, 1:10000) for 5 min. Cells were mounted on glass slide and images were examined and captured under a laser scanning confocal microscope (Olympus, FV1000).

### Data collection and statistic analysis

For quantitative data analysis, mean fluorescence intensity of immunoreactivity for iba1, proNGF, proBDNF, or MMP-9 was measured in six unit areas of each cell sample grew on coverslip and shown as mean ±S.E.M. (n=3-5 independent experiments) in immunocytochemistry. Percentages of PI-positive ones/total Hoechst-stained SH-SY5Y cells or TUNEL-positive ones/total DAPI-stained SH-SY5Y cells were counted and present in mean ±S.E.M. in bioassay. Intensity of immunoblotting bands in ratio to β-actin was also given as mean±S.E.M. (n=3, independent experiments) in western blotting. The differences between means were analyzed by one-way ANOVA (SPSS 18.0) with different markers as independent factors. When ANOVA test showed significant difference among means, the pair-wise comparisons between means were also performed by post hoc testing if required. The significance level was set at a *P* value of 0.05 for all analyses in this study.

## Results

### Activation of N9/BV2 microglial cells stimulated by LPS exposure in cell culture

**Figure 1:**
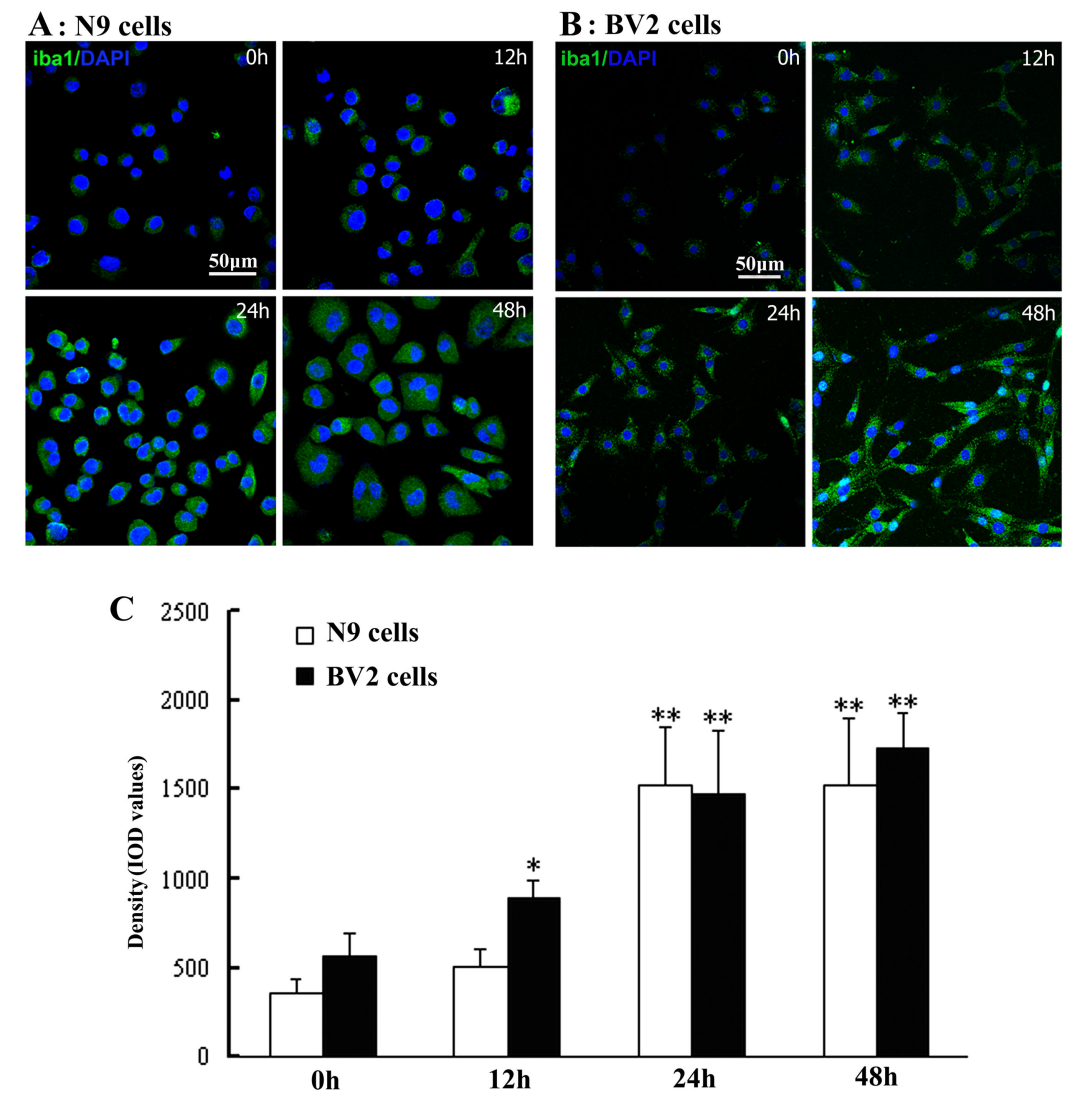
LPS-stimulated activation of microglial cells with increasing iba1-immunoreactivity in culture. The iba1-positive cells are shown in N9 cells (A) and BV2 cells (B) with DAPI counterstaining after LPS exposure of 12h-48h. Density in mean fluorescence intensity (MFI) of iba1-immunoreactivity among different N9 and BV2 cell groups is compared (C), ANOVA: * <0.05, ** <0.01 *vs* control.

By using iba1-immunostaining and morphological observation, activation of N9/BV2 microglial cells was detected following LPS exposure. Remarkable activation of microglial cells occurred at 24h and it was indicated with increasing iba1-immunoreactivity in N9/BV2 cells in culture (Figure 1A, B). DAPI Counterstaining showed that all (100%) N9/BV2 microglial cells exhibited iba1-immunoreactivity, and iba1-immunoreactivity was shown in N9 and BV2 cells of different time-points after LPS exposure. Quantitative analysis based on mean fluorescence intensity (MFI) indicated that significant increasing of iba1-immunoreactivity took place at 24h and 48h groups in both N9 and BV2 cells (Figure 1C).

### LPS-induced significant increase of proNGF expression in N9/BV2 microglial cells

**Figure 2:**
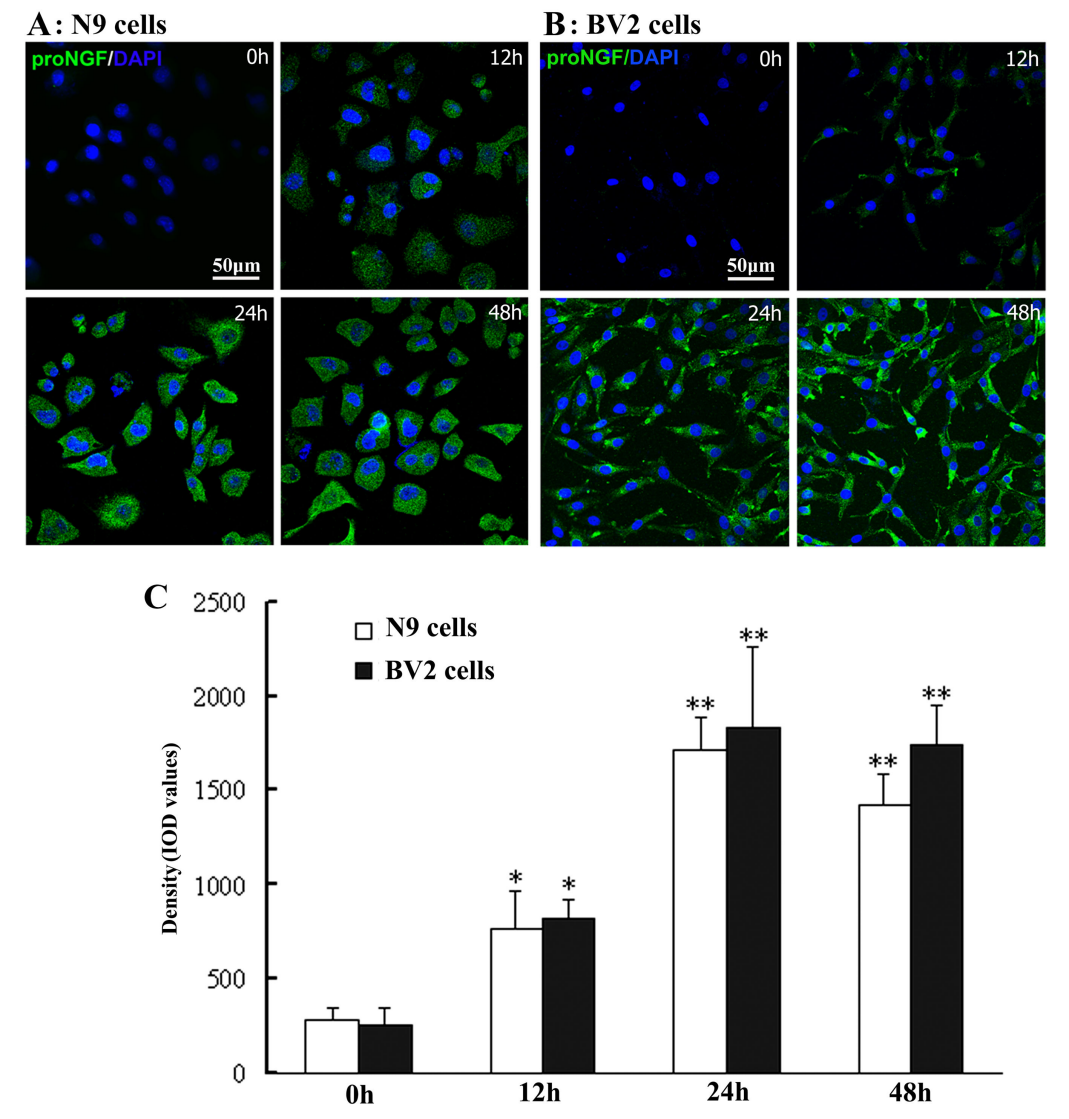
Increase of proNGF-immunoreactivity in N9/BV2 microglial cells after LPS stimulation. The proNGF-immunoreactivity is shown in N9 cells (A) and BV2 cells (B) with LPS exposure of 12h-48h. Density in proNGF-immunoreactivity (MFI) among different N9 and BV2 cell groups is compared (C), ANOVA: * <0.05, ** <0.01 *vs* control.

By immunofluorescence and laser scanning confocal microscopy, cellular localization or generation of proNGF were clearly identified in N9/BV2 microglial cells after LPS exposure. While it was hardly detected in control, proNGF-immunoreactivity was observed in both N9 and BV cells after LPS exposure. Slight weak but clear immunoreactvity was seen at 12h, strong immunostaining was seen at 24h and 48h after LPS exposure (Figure 2A, B). Data quantitative analysis showed significant increase of proNGF-immunoreactvity occurred at 12h, reached peak at 24h, and maintained high level after LPS exposure in both N9 and BV2 cells (Figure 2C).

**Figure 3:**
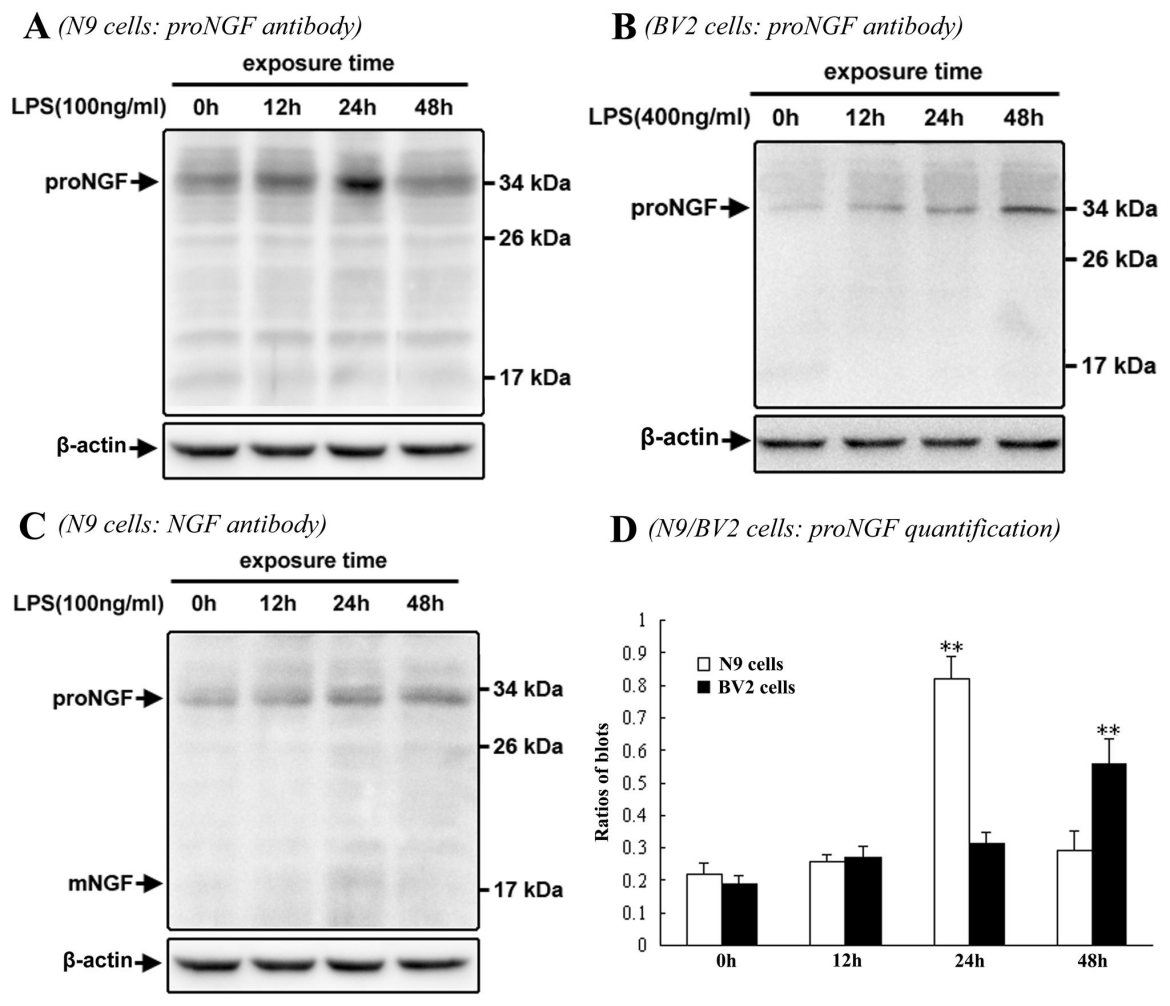
Western blot showing LPS-induced up-regulation of proNGF expression in N9/BV2 microglial cells. The immunoblot bands are respectively shown in N9 cells by proNGF antibody (A), BV2 cells by proNGF antibody (B), and N9 cells by NGF antibody (C). Comparison of proNGF levels is shown among distinct N9/BV2 cell groups (D). ANOVA: * <0.05, ** <0.01 *vs* control.

Western blot was further performed to confirm LPS-induced upregulation of proNGF expression in N9/BV2 microglial cells in different time-points of cell culture. Consistently, immunoblot band of proNGF was positively detected with anticipated molecular weight (about 34kDa) in both N9 and BV2 cells, and increase of proNGF expression was seen at 24h or 48h after LPS exposure (Figure 3A, B). Besides, in order to examine if any mature NGF was also produced in microglial cells, NGF antibody was applied for immunoblotting test and mature NGF (about 17kDa) was not detected in both control and LPS groups (Figure 3C). Quantitative data analysis indicated that significant increase of proNGF expression occurred at 24h or 48h after LPS exposure in N9 and BV2 cells in cell culture (Figure 3D).

### Expression patterns of proBDNF in N9/BV2 microglial cells in cell culture

**Figure 4:**
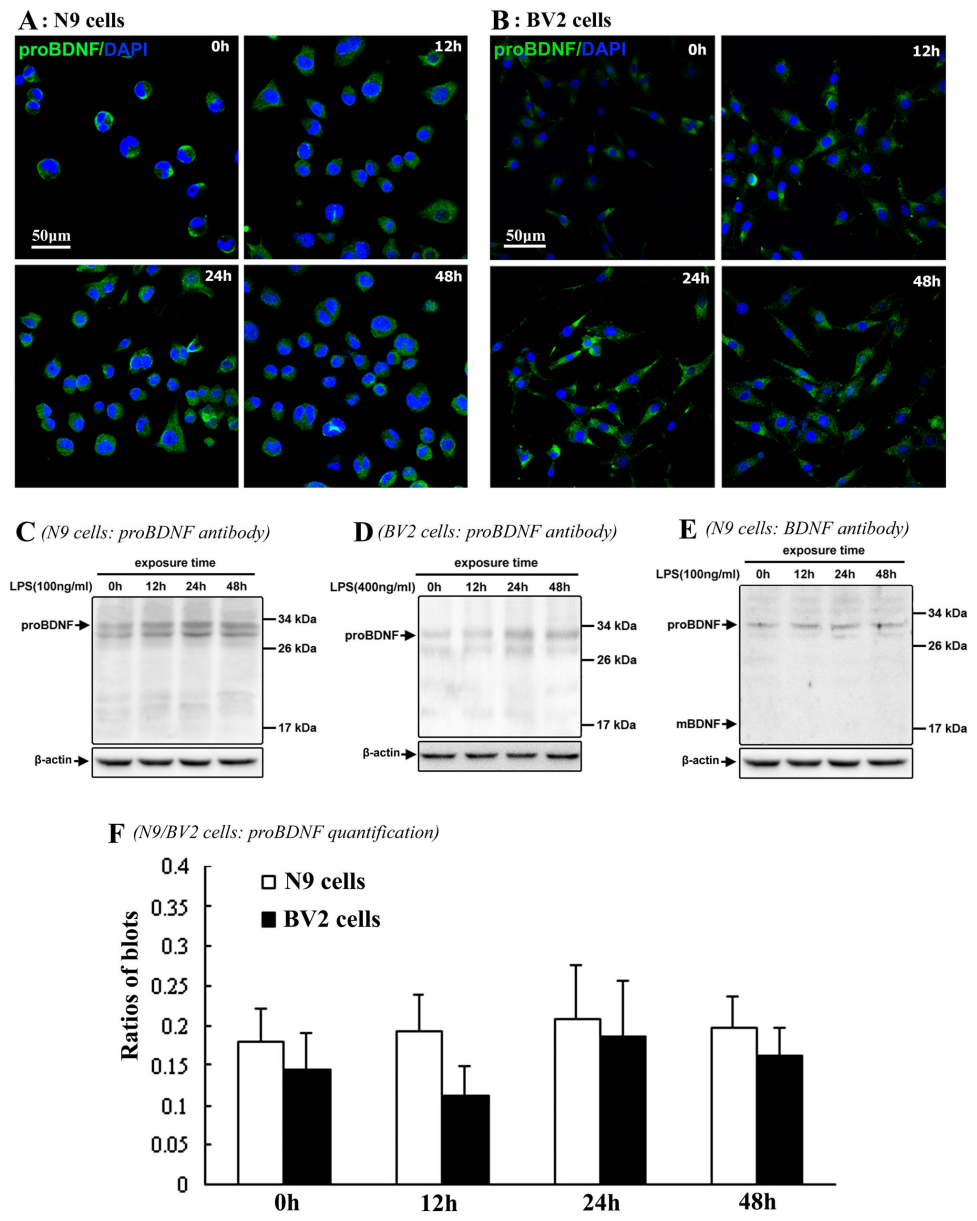
Expression patterns of proBDNF in N9/BV2 cells in both control and LPS exposure groups. Immunofluorescence shows proBDNF localization in N9 cells (A) and BV2 cells (B). Western blot shows proBDNF in N9 cells by proBDNF antibody (C), BV2 cells by proBDNF antibody (D), and N9 cells by BDNF antibody (E). Comparison of proBDNF expression levels shows no significant difference among distinct N9/BV2 cell groups (F).

Immunofluorescence and western blot were further applied to examine if proBDNF, another important one of pro-neurotrophins in the CNS, was also generated in the activated N9/BV2 microglial cells. The proBDNF-immunoreactivity was also observed in N9 and BV2 cells in control and LPS groups (Figure 4A, B). The immunoblot bands of proBDNF were positively observed in anticipated molecular weight (about 30kDa) in N9 and BV2 cells of both control and LPS group (Figure 4C, D). Besides, immunoblotting by BDNF antibody did not show any detectable mature BDNF (about 17kDa) in N9 cells (Figure 4E). Quantitative analysis did not show significant difference of proBDNF levels in distinct LPS groups in comparison with control (Figure 4F).

### Expression patterns of MMP-9 in N9/BV2 microglial cells in cell culture

**Figure 5:**
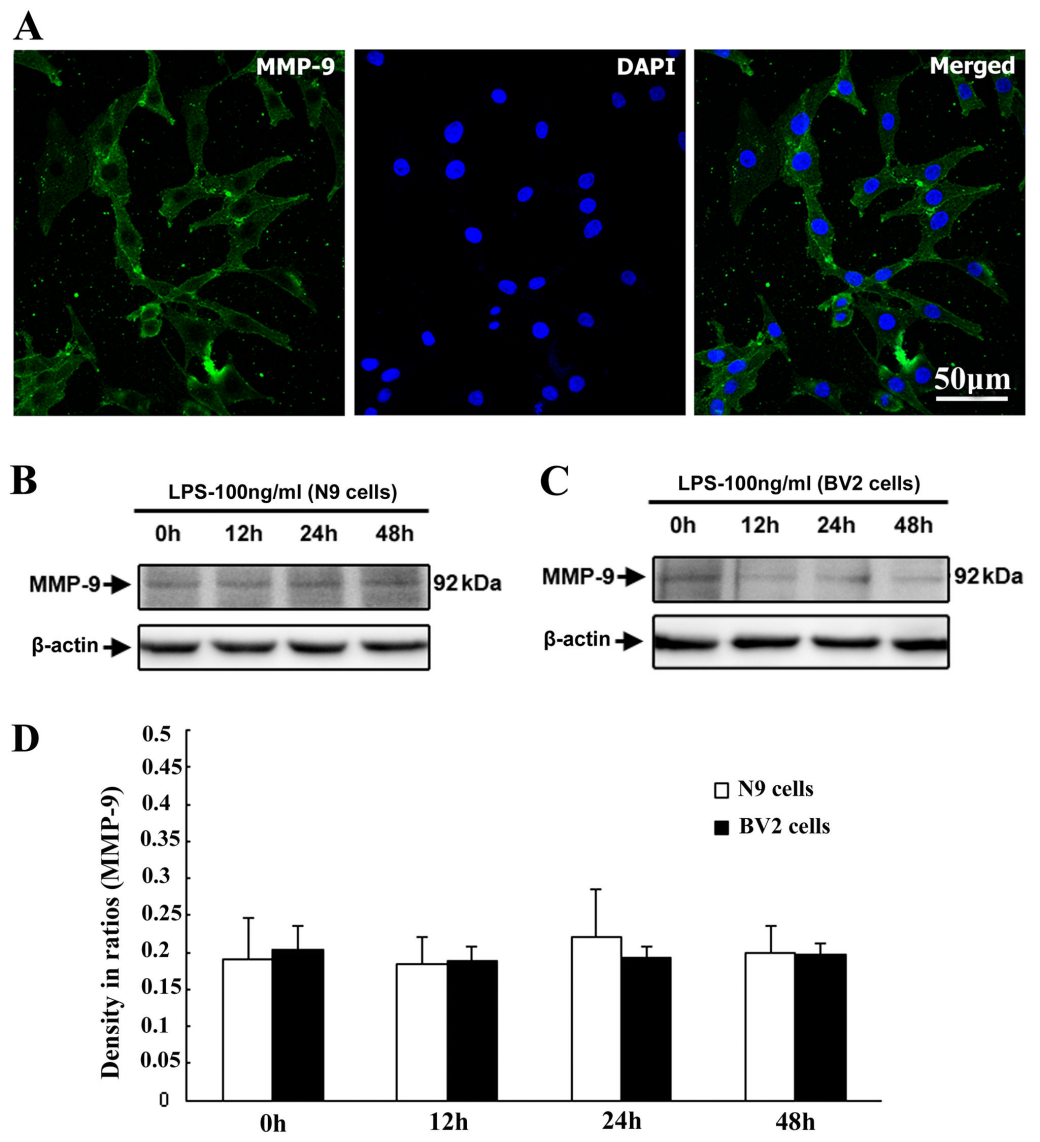
Expression patterns of MMP-9 in N9 and BV2 microglial cells in control and LPS exposure conditions. Immunofluorescence for MMP-9 is representatively shown in N9 cells of control group (A), immunoblotting bands of MMP-9 are shown in both N9 and BV2 cells of control and LPS groups (B, C). Comparison of MMP-9 expression levels does not show significant difference among distinct N9 and BV2 cell groups (D).

Immunofluorescence and western blot was also performed to detect expression of MMP-9, a key enzyme to function in degradation of mature NGF and neuroinflammation, in N9/BV2 cell culture. MMP-9 immunoreactvity was observed in BV2 cells of control and LPS group (Figure 5A). Western blot showed positive band of MMP-9 expression in both N9 and BV2 cells. Though slight increase of MMP-9 expression (about 92kDa) was seen in N9 cells after LPS exposure by immunoblot (Figure 5B, C), the quantitative analysis did not show significant difference of MMP-9 expression in both N9 and BV2 cells between control and LPS group statistically (Figure 5D). Data indicated that MMP-9 remained stable level under control and LPS stimulation conditions in both N9 and BV2 cells.

### Extracellular release or secretion of proNGF from N9 cells induced by LPS exposure

**Figure 6:**
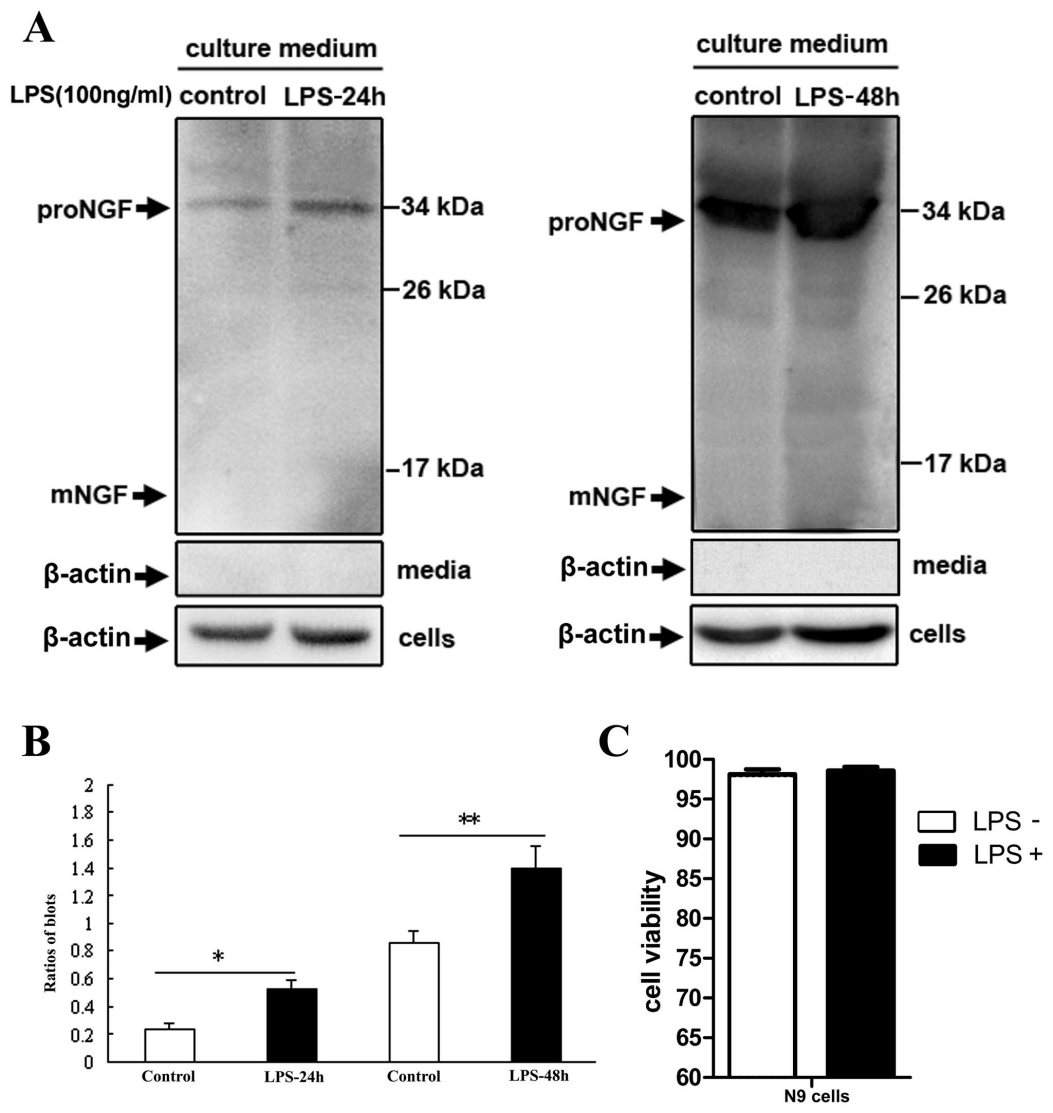
The release of proNGF from activated N9 microglial cells after LPS stimulation in cell culture. The proNGF is shown in culture medium of N9 cells of control and LPS groups (A), and increase of proNGF level is seen in LPS group (B). Cell viability by trypan blue test in N9 cells does not show difference between control and LPS group (C). ANOVA: * <0.05, ** <0.01 *vs* control.

To examine if proNGF directly release from microglial cells after LPS stimulation, culture medium or supernatant was collected and concentrated for immunoblotting detection. Western blotting clearly showed existence of proNGF in culture medium of N9 cells of both control and LPS group (Figure 6A). Significant increase of proNGF was detected after LPS exposure in cell culture (Figure 6B). However, β-actin was not detectable in culture medium of both control and LPS groups (Figure 5A).

In order to exclude any possibility that existence or increase of proNGF in culture medium might be resulted from cell cracking under LPS exposure, surviving and dying cells were further visualized by trypan blue staining in N9 cells of control and LPS group. It did no show significant difference of cell viability between control and LPS group (Figure 6C). Data thus indicated that extracellular release or secretion of proNGF did occur in microglial cells in response to LPS exposure or stimulation.

### Apoptotic cell death of SH-SY5Y cells induced by recombinant human proNGF

**Figure 7:**
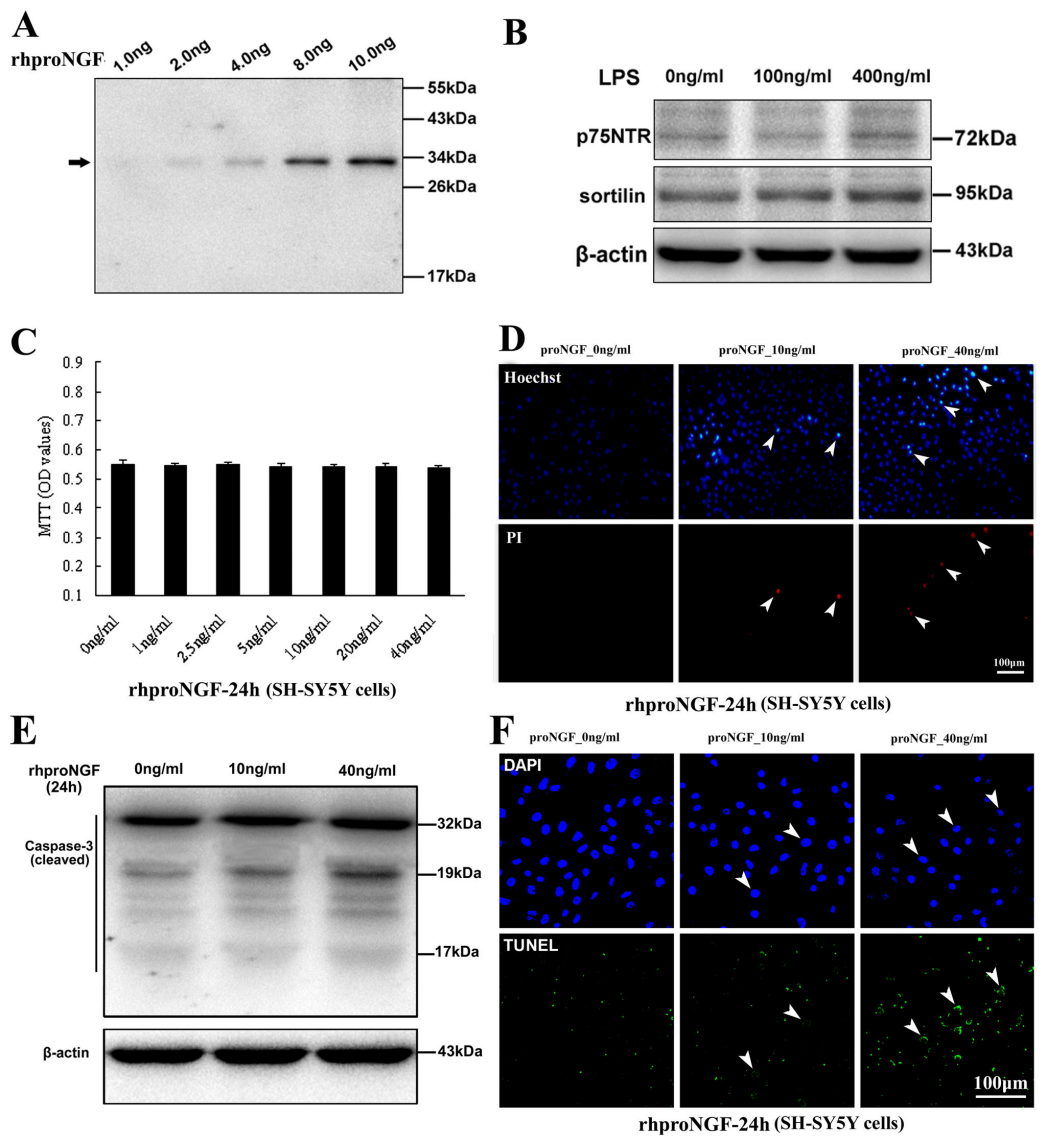
Bioassay of recombinant human proNGF (rhproNGF) in the SH-SY5Y cells. Characterization of rhproNGF is shown by western blot (A). Positive expression of p75NTR and sortilin is detected in SH-SY5Y cells in both control and LPS stimulation conditions (B). MTT shows no significant change of SH-SY5Y cell viability after proNGF treatment at different concentrations (C). Hoechst/PI staining indicates cell damage of SH-SY5Y cells resulted from proNGF treatment at 10ng/ml and 40ng/ml (D). E, Immunoblot shows a slight increase of cleaved caspase-3 expression (mainly 19kDa) following proNGF treatment (E). TUNEL assay shows appearance and increase of apoptotic cell death in SH-SY5Y cells with proNGF treatment (F).

A bioassay was further performed in SH-SY5Y cells to detect any effect of proNGF on cell viability or cell death by MTT assay, Hoechst/PI and TUNEL staining methods. Recombinant human proNGF (rhproNGF) was first characterized as molecular weight of about 34kDa by western blot (Figure 7A). Expression of p75NTR and sortilin, which could mediate proNGF-triggering apoptosis signaling and cell death [14,16,20], was positively detected in SH-SY5Y cells in both control and LPS exposure condition (Figure 7B). MTT assay did not show significant changes of SH-SY5Y cell viability after treatment of rhproNGF at different concentrations of 0, 1, 2.5, 5, 10, 20, 40ng/ml (Figure 7C). Hoechst/PI staining indicated some cell damage of SH-SY5Y cells occurred after proNGF treatment (Figure 7D), and cell count showed an increase of PI-positive cell percentage in groups with rhproNGF at 10ng/ml (1.5±0.8%) and 40ng/ml (6.8±1.9%) compared with control (0.05±0.03%). Western blot also showed a slight increase of cleaved (active) caspase-3 expression (remarkably at about 19kDa) following proNGF treatment of 40ng/ml (Figure 7E), and TUNEL assay further confirmed appearance of apoptotic cell death in SH-SY5Y cells with proNGF treatment (Figure 7F), and increase of apoptotic SH-SY5Y cells was seen in groups with rhproNGF at 10ng/ml (2.5±1.4%) and 40ng/ml (8.3±1.8% %) compared with control (0.6±0.3%).

## Discussion

Present study has demonstrated expression patterns and dynamic changes of proNGF, proBDNF and MMP-9 in N9 and BV2 microglial cells after LPS exposure in culture. New finding of this study is that LPS could stimulate increasing synthesis and direct extracellular release of proNGF in activated microglial cells. A bioassay in SH-SY5Y cells has further confirmed proNGF-induced apoptotic cell death by using MTT, Hoechst/PI and TUNEL staining methods. Taken together with proNGF functions in its triggering p75NTR-sortilin complex and initiating apoptosis signal, this study overall indicated that proNGF might appeal a new possible mechanism underlying aberrant microglial activation-mediated neurotoxicity in neuroinflammation state, also suggesting that proNGF may be a potential therapeutic target for the neurodegenerative diseases such as AD, PD and ALS [4,7].

It is well documented that proNGF preferentially bind to p75NTR receptor whereas mature NGF is preferred ligand for Trk receptor in the CNS [4,8]. Signals emanating from Trk promote neuronal survival and neurite outgrowth, while p75NTR-sortilin signaling might induce neuronal apoptosis and attenuate neurite outgrowth [15–22]. Moreover, functional interaction between p75NTR and Trk was demonstrated, in which proNGF induced PTEN via p75NTR to suppress Trk-mediated survival signal in brain neurons and proNGF also inhibited NGF-mediated Trk activation in PC12 cells [15,16]. Besides, the sortilin is also essential for neuronal cell apoptosis. As co-receptor of p75NTR, sortilin worked for proNGF and proBDNF effect in mediating neuronal apoptosis, and intracellular sortilin expression also regulated proNGF-inducing cell death during development [20,21].

Obviously, it is a critical question whether proNGF can be released from glial cells and actively function to trigger p75NTR-sortilin receptors in neurons. Until now, there have been few evidences on direct release or secretion of proNGF from activated glial cells including microglias and astrocytes. Most of previous studies concentrated on NGF production from reactive astrocytes in brains or diseases [26–28]. The reactive astrocytes predominately surrounded lesion neurons in AD brains and NGF secreted from reactive astrocytes induced cell death of p75-expressing motor neurons. Elevated NGF from reactive astrocyte was linked to degeneration of p75NTR-expressing motor neuron in transgenic ALS mice [14]. The reactive microglial cells might be another source of proNGF and this microglia-derived pro-NGF enhanced degeneration of photoreceptor cells in retina and apoptosis of motoneurons in CNS injury [26,27]. Pre-treatment with minocycline alleviated oligodendrocyte death possibly by inhibiting proNGF generation in reactive microglial cells during spinal cord injury [28]. We further revealed significant increase of proNGF synthesis in the N9 and BV2 microglial cells in response to exposure of LPS, which is well known to trigger microglial activation by binding Toll-like receptors.

More interestingly, the extracellular release or secretion of proNGF was identified in activated microglial cells with their response to LPS stimulation in this study. we also excluded the possibility that proNGF in culture medium was from cell cracking after LPS exposure by confirmation of trypan blue exclusion test and no detectable actin. In addition, MMP-9 was a key enzyme that may involve in NGF degradation and proNGF/NGF balance in neuroinflammation conditions [29,30], and pro-BDNF caused neuronal cell apoptosis by activating p75NTR-sortilin receptor as well [31]. Though expression of MMP-9 and proBDNF was positively observed in microglial cells, but no significant change was detected after LPS exposure. Thus, this study revealed an interesting finding that LPS stimulation could induce synthesis and secretion of proNGF in microglial cells, which might be underlying microglial neurotoxicity beside pre-inflammatory cytokines they may release in response to stimulation [2–4].

Though LPS-induced increase of proNGF synthesis was clearly shown with undetectable NGF in N9 and BV2 microglial cells, it still remains unclear what key mechanism implicating in LPS-induced proNGF production. Impaired balance of proNGF and NGF was induced by peroxynitrite in Diabetes, which then causes neurovascular injury [32]. Decreased serum levels of mature BDNF, but not proBDNF, was found in patients with depressive disorder [33]. Imbalance of proNGF/NGF generation or function might trigger learning and memory deficits and neurodegeneration in the transgenic mouse model [34]. Previous evidence showed that MMP-9 was responsible for degradation of NGF under neuroinflammatory condition and might influence balance of proNGF and mature NGF level [30]. MMP-9 was also involved in BV-2 microglial cell migration and expression of MMP-9 was elevated in spinal cord in a mouse model of ALS [35,36]. Besides, MMP-7 was found to regulate proNGF cleavage under kainic acid insult while MMP-1 functioned in Hella cells and MMP-8 acted in progenitor cell migration and recruitment into atherosclerotic lesions [37–39]. By western blot in this study, we examined MMP-9 expression and no significant change of MMP-9 level was detected after LPS stimulation. In addition, it is known that proprotein convertase such as furin is theoretically able to convert proNGF into NGF. Proprotein convertase furin enhanced survival and migration of vascular smooth muscle cells though processing of proNGF [40]. If furin also took action in N9 and BV2 microglial cells, increase of NGF would be observed along with proNGF increase after LPS stimulation under a stable MMP-9 condition. In this study, however, LPS-induced increase of proNGF level was observed in comparison with that of control, while the amount of NGF was undetectable in both control and LPS-stimulated conditions. Our data thus suggest that proNGF increase might be most possibly resulted from LPS-triggered intracellular signaling pathway related to proNGF synthesis in these activated microglial cells, which shall need further investigation.

Finally, a growing line of evidences have shown that dynamic changes of proNGF, p75NTR and sortilin might be involved in aging-related neurodegeneration, disease onset and progression of the degenerative diseases [9–14,41–45]. For instance, increase of proNGF and sortilin expression was demonstrated in the age-related neurodegeneration and brain injury conditions [41,42]. The proNGF-p75NTR-sortilin complex also mediated death of Natural Killer cells and dorsal root ganglion neurons after injury [43,44]. Impaired balance of proNGF and NGF was involved in neurovascular injury in diabetes, and abnormal upregulation of proNGF, p75NTR and sortilin was also associated with retrovirus-induced spongiform encephalomyelopathy [32,45]. In AD condition, proNGF produced in the brains elicited apoptosis of basal forebrain neurons with p75NTR expression, and proNGF purified from AD human brains induced neuronal apoptosis by binding p75NTR receptor in neuronal culture [5]. Imbalance of proNGF/NGF seemed a cause for vulnerability of cholinergic neurons and susceptibility to late-onset in AD [10–12]. In ALS, both pro-form and mature of NGF could activate p75NTR and induce apoptosis of motor neurons, and p75NTR antagonist improved progression in ALS disease in transgenic mouse model [13,14]. Our recent observation with LPS animal model showed abundant microglial activation and up-regulation of proNGF, which might be involved in neuronal degeneration in the substantia nigra (personal unpublished data), supporting our point that proNGF-p75NTR-sortilin signaling might play certain role in pathogenesis and disease progression of PD [4]. Therefore, new therapeutic strategy will hopefully be developed by targeting on proNGF-p75NTR-sortilin signaling in aberrant activated microglias for treatment of various neurodegenerative diseases in human beings.

## Conclusion

This study has revealed significant increasing synthesis and direct extracellular release of proNGF in activated N9/BV2 microglial cells induced by LPS stimulation. The proNGF-inducing apoptotic cell death of SH-SY5Y cells was further confirmed by a bioassay. Data of this study overall may appeal a new pathway or mechanism underlying microglial toxicity in the neuroinflammation and a potential target for therapeutic manipulation of the neurodegenerative diseases.
